# From the tobacco industry's uses of science for public relations purposes to the alcohol industry: Tobacco industry documents study

**DOI:** 10.1111/dar.13649

**Published:** 2023-03-18

**Authors:** Andrew Bartlett, Jack Garry, Jim McCambridge

**Affiliations:** ^1^ Department of Health Sciences Seebohm Rowntree Building, University of York York UK

**Keywords:** alcohol industry, alcohol science, public health, tobacco documents

## Abstract

**Introduction:**

Associates for Research in Substances of Enjoyment (ARISE) was formed by tobacco companies in the late 1980s designed to counter public health policy development. This study examines the alcohol content of ARISE and the contribution of ARISE to alcohol industry activities in a key period in the globalisation of the alcohol industry, generating insights into the inter‐relationships between the tobacco and alcohol industries in their involvements in policy‐oriented science.

**Methods:**

We systematically searched the UCSF Truth Tobacco Documents Library for information about ARISE, alcohol and the alcohol industry. This material was supplemented with an analysis of the contributions by ARISE associates to one volume in the International Center for Alcohol Policies (ICAP) book series on alcohol and pleasure.

**Results:**

ARISE placed nicotine alongside caffeine, chocolate and other foods, and alcohol as treats which brought pleasure and other benefits. Alcohol was thus intrinsic to the ARISE project for the tobacco industry. This study shows that at a formative moment in the mid‐1990s the major alcohol companies took advantage of the intellectual inheritance and personnel provided by the tobacco industry when establishing ICAP. Key to this was an ICAP conference that resulted in *Alcohol and pleasure: A health perspective* (1999).

**Discussion and Conclusions:**

Not only did ARISE use alcohol to play a supporting role in a sophisticated tobacco industry strategy, the alcohol industry engaged with ARISE as part of its own strategy. This shows the importance of careful attention to corporate activities on the fringes of peer‐reviewed science.

## 1 INTRODUCTION

There has been no shortage of reasons for concern, but little substantive tradition of formal study of the nature of alcohol industry involvement in science [1, 2, 3]. This has begun to change recently as the scale of the involvement has become more apparent [4, 5, 6, 7, 8]. Related strands of work have directly examined alcohol industry scientific interventions within [9] and outside [10] peer‐reviewed journals. Attention has been given to scientific topics of interest to industry actors, such as the putative cardiovascular benefits of low dose alcohol consumption [11, 12, 13] and alcohol and violence [10].

The Master Settlement Agreement, which mandated the release of some industry documents, has allowed the development of an in‐depth understanding of the internal workings of the tobacco industry [14]. Management of the science which showed their products to be harmful was foundational to the tobacco companies' long‐term public relations (PR) strategy, initially led by the PR firm Hill and Knowlton from the early 1950s onwards [15, 16]. The first tobacco industry‐sponsored book appeared in 1960, producing positive news coverage appreciated within the industry [17]. By the 1970s, the management of science by the tobacco industry included the recruitment of social scientists and others with covert funding in order to generate controversies on the benefits of smoking, and to cast doubt on the attendant social and health costs [18]. The modus operandi into the 1980s involved ‘cross‐cultural research’ undertaken in different disciplines as well as countries, third‐party organised academic conferences with speakers chosen by tobacco companies, and third‐party book projects with undisclosed author connections to tobacco [17]. These activities emphasised the positive social roles of tobacco and sought to both influence public opinion and defeat public health policy developments contrary to industry interests.

The 1988 US Surgeon General report which concluded that nicotine was addictive made addiction a key scientific issue for the tobacco industry, prompting multiple companies to establish Associates for Research in Substances of Enjoyment (ARISE) [17, 19]. This was a network of scientists led by David Warburton, a tobacco‐funded psychologist who viewed nicotine as non‐addictive and performance enhancing [17, 19]. ARISE held international events biennially: Florence 1989 (predating the naming of the group as ARISE in 1990); Venice 1991; Brussels 1993; Amsterdam 1995; Rome 1997; and Kyoto 1999 [17, 19]. Three edited collections with chapters based on these conferences were published: *Addiction Controversies* in 1990 (from Florence 1989) [20]; *Pleasure: The Politics and the Reality* in 1994 (from Venice 1991) [21]; and *Pleasure and the Quality of Life* in 1996 (from Brussels 1993) [17, 22]. All three were edited by Warburton, the final book in partnership with Neil Sherwood. The books were ostensibly aimed at scientific audiences and in 1994, at the behest of a tobacco company, the word ‘substance’ was dropped from the ARISE acronym in favour of ‘science’ [17].

Tobacco companies were involved in the organisation and funding of the events and the associated international PR strategy was co‐ordinated by PR company Fishburn Hedges from London [17, 19]. As well as the books, opinion polls and other press release materials were widely disseminated, particularly via a core group of ARISE ‘associates’ serving as spokespersons [19]. The British tobacco company Rothmans was prominent in managing the operation, along with Philip Morris, though other tobacco companies were also involved [17, 19]. ARISE was UK‐led, with Smith [19] for example identifying more than 40% of participants, one‐third of spokespersons and ~30% of all media articles as UK based.

The Master Settlement release of documents has also shed some light on the alcohol industry. Work based on the tobacco industry documents archive has identified long‐term PR programs by the alcohol industry to influence science [23]. These were originally co‐designed with PR company Hill and Knowlton, who had worked with the US distilled spirits industry *before* working with tobacco companies [23]. Alcohol and tobacco industry interactions in PR strategy development later involved key personnel moving between sectors [23]. The basic features of this alcohol PR approach appear highly stable over many decades, perhaps because it was both undetected—or at least uncontroversial—and successful in securing its goals. In the guise of the pursuit of the public good, the interests of the industry have been secured by the creation of an international network of national level ‘social aspects’ organisations, beginning with the Portman Group in Britain [23]. The global‐level counterpart was the International Center for Alcohol Policies (ICAP) [24]. Such organisations appear to be a key component of wider alcohol industry political strategies [25, 26, 27, 28, 29].

The tobacco industry documents archive has also revealed the control Philip Morris exercised over the wholly owned Miller Brewing Company, and the ways in which this facilitated influence in the US brewing trade associations and in the formation of ICAP. Studies have shown that tobacco companies targeted different sectors in building their constituencies and that a division of labour was agreed between tobacco and alcohol industry organisations in opposing excise tax increases in the US, with the alcohol industry not simply operating as a subordinate of the tobacco industry [30].

Jernigan [24] identified ICAP as an attempt by major alcohol companies to counter the World Health Organization. ICAP was formed in 1995 in the early days of the transformation of the global alcohol industry [31]. ICAP recruited Marcus Grant from the World Health Organization to lead the organisation, and its activities aroused concern within the scientific community [32, 33, 34, 35, 36]. It was nonetheless successful in recruiting scientists to work with it [7], and while Jernigan [24] has analysed the breadth of the public‐facing activities, there is little secure in depth understanding of its formative influences, other than on the involvement of Philip Morris [37].

Both Landman et al. [17] and Smith [19] have investigated ARISE in depth, with tobacco industry management of their activities a principal object of study. This article extends what is known about alcohol industry involvement in science, using the UCSF tobacco industry documents archive as the primary data source. Existing studies examine certain aspects of ARISE ideas and media impacts in some depth, and the intention here is not to retrace this ground. The key questions of this article are; in which ways were the alcohol industry and alcohol as a commodity involved in the ARISE project; and how did such involvements contribute to the development of alcohol industry political and scientific strategies?

## 2 METHODS

Methods for searching, collecting, and analysing the documents in the UCSF Tobacco Industry Documents library have developed over time [38, 39]. We used a snowball technique, with early searches for scoping purposes followed up by searches in pursuit of the most promising lines of enquiry. For example, we searched by keywords including ARISE (and its various combinations), the names of known ‘associates’ and of events and publications. Using the search facility, Jack Garry performed the initial and subsequent waves of searches. After screening by Jack Garry, the documents were read by Jim McCambridge, who reduced the dataset for focused study, and further searches were undertaken. The searches were part of a larger project to identify material by which to better understand the alcohol industry. The analysis draws heavily on accounts internal to ARISE and the tobacco and alcohol industries, and depends upon the sources cited in this article. This material was checked and triangulated with publicly available information to appraise the validity of the content, with themes developed by all three authors.

This work was complemented by an examination of *Alcohol and pleasure* [40], in particular the chapters written by ARISE associates [41, 42, 43, 44]. This book was part of the ICAP book series, published by Routledge, and was the result of an ICAP conference in which David Warburton played an organising role. This material is a clear‐cut example of direct collaboration between the alcohol industry and ARISE, and an analysis of the framing of these chapters provides insight into the purpose and function [45] of ARISE's program to the alcohol industry.

## 3 RESULTS

### 3.1 The roles of alcohol in the evolution of ARISE

ARISE owes its conception to the conference in Florence in 1989 on ‘Comparative Substance Use’. From the beginning, alcohol was on the agenda, as the minutes of the 1990 meeting in Zurich—at which the name of the organisation was agreed—make clear [46]. A distinction was made between “legal, enjoyed substances”—for which they used the German word 'Genussmittel', translated as ‘treats’—and ‘socially unacceptable substances’. While Warburton describes ARISE as a continuation of the work of the Florence conference [49] (which was a credible contribution to scientific discourse), ARISE restricted its attention to commodities such as tea, coffee, chocolate, cigarettes and alcohol (when used in ‘moderation’).

ARISE was to function as a counter to public health narratives with a focus on *harm* by developing a narrative of the *pleasure* of consumption. The ARISE acronym was justified as it ‘epitomises our feeling that there should be some resurgence against the Calvinistic attack on people obtaining pleasure from substances and on their freedom of choice to do so’ [46]. Notwithstanding the injunction that ARISE ‘should be apolitical as a group and act as independent scientists’, these minutes of the 1990 meeting recognised that ‘Associates’ would ‘advise on legislative reports’ and ‘make constructive statements on legislative proposals’ [46]. That the intellectual program would find practical, political application was clear.

The tobacco industry origins of ARISE are well established. A 1994 presentation explicitly described it as an industry response to the US Surgeon General's claim that ‘nicotine was as addictive as heroin or cocaine’, and that ‘a group of academics was identified and called together to’ ‘review the science of substance abuse’ [47]. This presentation also listed Guinness and Miller as ‘past or present supporters’, alongside Nestle, Kraft, the European Advertising Agencies Association and Coca Cola.

ARISE was explicit as to whom it was opposed; a 1991 press release described ARISE as facing off against ‘Health Lobbyists’ who attack all the pleasurable substances, while at the same time insisting that ARISE is ‘in no sense a lobby group’ [48]. According to the press release, evidence of ‘the flimsiest kind’ attributing harm to pleasurable substances is accepted because it ‘conforms with one's moral righteousness’, while evidence of benefits is ‘suppressed, turned upside down, ridiculed and dismissed’ [48]. Framing public health science in this manner provides a scientific and *political* rationale for ARISE.

After Florence, the meetings were presented as workshops [49], looser and arguably more ambiguous a label than ‘scientific conference’. These workshops had many of the same people, the ARISE ‘associates’, presenting. It is notable that while the *Addiction Controversies* [50] drew in a wide range of figures who were, or would become, leading addiction scientists, the second book, *Pleasure, the Politics and the Reality* [21] largely comprises contributors from the core group of key ARISE figures. Warburton's introduction claims ‘we want to develop a balanced perspective on the use of pleasurable substances’ [21, p. 1], which prefigures the ICAP theme of ‘balance’, in both cases understood as providing a counterweight to public health research.

The ARISE narrative was not just that consumption of pleasurable substances brought benefits, but that there were psychological and psychosomatic burdens resulting from viewing such ‘treats’—including ‘moderate’ alcohol use—as harmful [32]. Alcohol, and public health attention to its consumption, was thus an integral component of the ARISE project. Psychologists, including Warburton and other specialists in the field of psychopharmacology were prominent, along with some medical figures.

The fundamental importance of PR to the project is laid bare in the overseas agency brief prepared by Fishburn Hedges in 1994 [51], where the ARISE objectives are stated as; ‘to establish ARISE as a recognised, credible and permanent international network of scientists, academics, journalists and supporters’ [51]. Brewers are identified among the supporters. In 1994/95 they aspire; ‘to conduct a more organised and proactive campaign to ensure its views are heard and recognised by international opinion formers’ [51]. It is important to note the opinion formers in question were not scientists. The thrust of ARISE was not a genuine attempt to engage in the constitutive forum of science [52], but to change the opinions of journalists, the public and policy makers *about* science. For example, ARISE produced survey data for press releases rather than peer‐reviewed reports [53], while surveillance of media impact was a key measure by which the success of ARISE was reported [54].

Alcohol as a substance had always been part of ARISE's remit, though the focus on particular products and segments of the industry changed over time. While distilled spirits were part of the ARISE narrative at the time of the Venice conference [48] and the alcohol industry was listed as one of the sponsors of the event, later meetings narrowed the focus to beer and wine, with only brewers identified as funders. As we have seen, by 1994 Guinness and Miller were listed among the past or present supporters of ARISE [47], and both were key to the emergence of global alcohol industry political strategies [23, 37]. ARISE output was used directly by the industry; for example, there is much ARISE content in the Guinness magazine ‘Perspectives’ on alcohol science and policy from 1990/91 [55], which foreshadows later ICAP content. By that time Guinness had been instrumental in the formation of the Portman Group [13]. Alcohol industry involvement in ARISE provided a resource that could potentially be called upon within what were the formative years of globalising alcohol industry strategies, including by ICAP.

Much ARISE work on alcohol was produced by Geoff Lowe, a UK psychologist. Lowe was known to the tobacco industry, having had an application to the Council for Tobacco Research turned down in 1974 [56]. His work included attention to the effects of tobacco and alcohol when combined [57] and ranged from experimental studies [57] to qualitative analyses of the use of pleasurable substances, including alcohol, in the Mass Observation study [58]. He explored a range of standard ARISE and tobacco industry themes including stress relief, a subject of longstanding tobacco funding [59], and creativity (relating to both alcohol and tobacco) [60, 61]. That Lowe's ARISE‐linked scientific work on alcohol was largely not published in peer‐reviewed journals was in keeping with the ARISE strategy; ‘science’ and ‘expertise’ mattered to ARISE not because it was a contribution to ongoing debates in scientific fora, but because having alcohol content authored by an academic legitimated the ARISE PR narrative. That Lowe, along with other key players in ARISE, was able to transition to working with ICAP may be the most significant proximal aspect of ARISE's contribution to alcohol industry involvement in science, more so than the modest alcohol content of ARISE itself. The transmission of expertise in recruiting scientists into networks managed (directly or indirectly) by industry is a more distal, though important, legacy of the tobacco industry's decades of involvement in science to organisation of alcohol industry scientific programs.

### 3.2 ARISE, ICAP and ‘Alcohol and pleasure’

In 1998, ICAP began publishing the ‘International Center for Alcohol Policies Series on Alcohol and Society’ through Routledge. In format, they were much like the ARISE books edited by Warburton, consisting mainly of edited collections. There were also important differences, with many chapters written by prominent academics working on various aspects of alcohol science, alongside contributions produced by, or in collaboration with, alcohol industry employees and/or the staff of industry‐funded social aspects.

Early in the ICAP book series, but late in the life of ARISE, ICAP published *Alcohol and pleasure* [40], a book with a substantial ARISE contribution. Warburton and Lowe, and ARISE associates John Luik and Jan Snel contributed chapters to the book (though only Warburton listed his connection to ARISE), based on a 1998 conference in New York. Warburton was on the 8‐person advisory board organising the conference, through the resulting book was edited by Grant and Stanton Peele, an American psychologist who argued against the medical model of addiction. In the preface to the book, Grant, identifies this event as being conceived at an ICAP board meeting in 1996.

This places the genesis of the book shortly after the 1995 ARISE workshop ‘Living is More Than Surviving’ [62], attended by all four of the ARISE authors in *Alcohol and pleasure*. Warburton, Luik and Snel wrote the summary of the meeting. At that workshop, it was claimed that:

‘The New Puritanism has become the ideology of the late 20th century and has replaced more traditional ways of thinking about individuals, their relations to each other, society and, most particularly, pleasure'. [69, pp. 5–6].

This framing is a further development of the earlier themes of ARISE, and is echoed, at least in parts, by many chapters of *Alcohol and pleasure*.

Warburton and Luik present chapters 1 [41] and 2 [42] in the book respectively, and both recycle generic ARISE themes. Warburton incorporates a brief discussion on alcohol and mood and otherwise sets the scene by presenting the ARISE basic perspective [41]. He introduces readers to ideas such as ‘pleasure inoculation’, that pleasure can be constitutive of good health as improved mood leads to improved immune response [41, p. 16]. He also sets out ARISE's political function:

‘The medical evidence that pleasure is good for people is a useful riposte to the moralistic self‐righteousness of those who believe there is only one way to live life—denial'. [41, p. 20].

To whom is this work ‘useful’ is left unstated.

Luik's chapter offers a purported history of pleasure [42]. This features the alleged constraints of Christianity, the intents of the World Health Organization, fundamental problems in health promotion and the tyranny of science, and positions public health initiatives as part of ‘a radical assault on what it means to be a free person in a democratic society’ [42, p. 29]. Echoing an ARISE theme, Luik claims that ‘… health promotion passes itself off as scientific’ [42, p. 39]. Luik undermines the scientific legitimacy of public health research, while presenting organisations such as ARISE and ICAP as, in contrast, taking up the duty of providing the public with ‘rigorously objective scientific information’ [42, p. 35].

Luik, a philosopher within ARISE [62, 63], was a controversial character—well known to tobacco industry interests and dismissed twice from Canadian academic institutions for misrepresenting his credentials—his major themes included the corruption of science and policy by public health interests. His potential for contributing on alcohol had earlier been spotted by Samuel D. Chilcote, Director of the Tobacco Institute and a key figure linking the two sectors, having been previously employed by the alcohol industry [13]. In 1993, along with the names of tobacco company contacts, Chilcote [64] passed an article by Luik to Morris Chafetz, the founding director of the National Institute on Alcohol Abuse and Alcoholism who had gone on to receive alcohol industry funding for a foundation he established [65]. There can have been little doubt on the part of ICAP about the nature of the contribution to be expected from Warburton, Luik and the other ARISE associates.

The other two ARISE chapters are less polemical, located deeper in the body of the edited volume. Lowe, as the alcohol specialist within ARISE, is an unsurprising inclusion. This chapter is focused on alcohol and discusses drinking over the lifecourse [43]. The material includes arguments seen in other alcohol industry initiatives, notably throughout the ICAP book series, that drinking is a skilled activity that needs to be correctly learned to optimise pleasure. For Lowe, skilled drinking is about people learning ‘to develop their consumption so that they develop a repertoire of drinking and ingestion styles to be used on different occasions and for different purposes in different contexts’. ‘As with many other skilled behaviours—sports, cooking, musical skills and so on—the more skilled the practitioner, the higher the degree of pleasure and enjoyment [43, p. 259]. Seen through the lens of ARISE, as set out by Warburton in the opening chapter, skilful drinking connects to health in two ways—first by ensuring that drinking is appropriate to context, and second by enhancing pleasure. According to Lowe:

‘Although it is highly likely that some pleasurable substances are, in some circumstances, be really bad for us [sic], it is even more likely that enjoyable pleasures really are good for us'. [43, p. 257].

On the other hand, Snel is a surprising inclusion and arguably the content of the chapter somewhat more surprising still. Snel spent his career at the University of Amsterdam working mainly on caffeine (among the list of past and present ARISE ‘supporters’ in 1994 was the Coffee Science Information Center), the subject of his ARISE presentations in 1995 and 1997. Snel had no track record of alcohol research. The chapter emphasises the functional value, as well as the pleasure, to be gained by drinking alcohol responsibly, and also critiques the alcohol research literature; in that being focused on problems and alcoholism the literature is biased to see alcohol only in terms of harms [44]. He wrote:

‘The preponderance of alcohol research creates the impression that alcohol is a substance that has only harmful effects on people's health and cognition, and that drinking must lead eventually to addiction. Thus, if people accepted the opinions of many health scientists, they would decide that alcohol is a poison that should be banned’. [p. 278],

and yet, ‘Both pleasure and moderate use have been proven to be healthy’ [44, p. 277]. This constitutes a version of the basic ARISE anti‐public health narrative, as applied to alcohol and alcohol research. To some extent Snel goes even further, suggesting that alcohol is part of an ‘optimising lifestyle’, writing that:

'research on the effects of alcohol on cognitive functioning and stress reduction indicates that alcohol is a functional, useful component of lifestyle. Moreover, the pleasure derived from responsible drinking is an important means to achieve an optimum state'. [44, p. 277].

The direct link between ARISE and ICAP was established towards the end of ARISE's existence and in the early years of ICAP. The ICAP meeting at which it was agreed to host the conference from which *Alcohol and pleasure* were derived was held in 1996, the year after ICAP's formation, while the book itself was published in 1999, the third book in the 10 book series which ran from 1998 to 2010. By the time of ARISE's Kyoto event in 1999, of the core group only Warburton and Snel remained on the organising committee [66]. Landman [17] could find no further information on ARISE and wondered why it disbanded when having served industry so well. Smith [19] reports that the tobacco industry had stopped funding Warburton by 2001, by which time he had replicated the ARISE model for a wine company; promoting a survey that purported to identify something called ‘kitchen performance anxiety’. This did not make it into the peer‐reviewed literature. As far as the alcohol industry was concerned, however, the ICAP book series, to which ARISE made a significant if fleeting contribution, represented a determined and sustained attempt to shape the scientific discourse around alcohol—an open and explicit attempt to shift the paradigm—which continued until the final book in 2010. While ARISE is now long defunct, ICAP merged with the Global Alcohol Producers Group in 2014, and shortly after was rebranded as the still existing International Alliance for Responsible Drinking.

## 4 DISCUSSION

This study shows that at a key formative moment the major alcohol companies took advantage of the intellectual inheritance provided by the tobacco companies in the form of ARISE to support their emerging global political strategy in ICAP. Alcohol companies were aware of ARISE and indeed had been direct sponsors. ICAP might have sought to promote other benefits of alcohol, and around that time alcohol companies were investing in the funding of cardiovascular research apparently showing physiological benefits [11], yet ICAP chose to do a book on *pleasure*. This complemented existing work that was explicitly intended to shift the paradigm away from whole‐population studies of harm to research of ‘drinking patterns’, including ‘healthful’ drinking.

The connections between the alcohol and tobacco industries in both PR and attempts to influence and shape science for that purpose run well beyond ARISE [23]. Warburton himself was involved in other tobacco‐created scientific networks that also included alcohol. For example, the Philip Morris‐funded projects Sunrise and Cosmic included alcohol alongside other substance use, and these pre‐dated ARISE and ran alongside it [67, 68, 69]. In those examples, there was a similar emphasis on networks, though their activities largely used research grant funding as the glue that connected the networks rather than attendance at events or co‐publications. There were also other key figures such as the Yale historian David Musto, who provided Philip Morris with projects they paid for specifically on alcohol [67, 69, 70]. In this context, ARISE is a well‐described ‘case’ through which we may understand other industry scientific programs, with organisational as well as intellectual qualities that are portable across corporate sectors facing similar policy challenges. As a result of the Master Settlement Agreement and the existence of the UCSF library, we are able to know more about the relationship between ARISE and the alcohol industry than we will know in other such cases of transfer across sectors.

Although ARISE was one venture in the longer history of tobacco industry corruption of science, the operating model of drawing in other related sectors, also contained within it the possibility that alcohol or food for example could at key moments draw on the arguments and personnel mobilised by ARISE and the tobacco companies as a resource. Companies such as Philip Morris, which owned both Kraft and Miller Brewing, provided a complementary and more direct means of transmission of key ideas and personnel. This is what appears to have been done in the case of the link between ICAP and ARISE, though it should be noted that ICAP and its sponsors ultimately went in a different direction from the ARISE project, engaging in a much more serious attempt to influence the content of science, as indeed the tobacco industry had done for decades. The alcohol industry was not content to restrict the role of ICAP to that of a PR device, but drew on the longer tobacco industry experience of shaping science. ICAP was formed at a moment of scientific opportunity in the mid‐1990s, when attention to drinking patterns and harm reduction ideas were influential. The tobacco and alcohol industries continue to collaborate to the present day in influencing science, and how scientific evidence is used in policymaking [71].

As the 1990s ended, depositions identifying ARISE (e.g., [72]) were being made in US legal cases, and it may be that having been so identified, ARISE had outlived its usefulness to the tobacco industry. Without new people, money, ideas or research, the propaganda machine may also have got bored listening to itself. There may, however, be enduring lessons in the way in which both the tobacco and alcohol industries cultivated scientists from domains beyond biomedicine, with the recruitment of psychologists and other social scientists into both ARISE and ICAP scientific programs. The value of comparative substance use projects, and crossovers with gambling, indicates that the corporate sectors which produce addictive products operate with sophisticated high‐level approaches to their own businesses that include managing the addiction scientific field, which is still largely working in silos [73].

ICAP appears to have continued to work effectively for the major alcohol companies, delaying the introduction of alcohol policies across the world, in similar ways to those pioneered by the tobacco companies, after the demise of ARISE [74]. In so doing, ICAP created a range of books, reports, documents and other artefacts that provide a basis for careful study, until its demise at the end of 2013, interestingly, following Jernigan's dissection of the activities of ICAP the previous year [24]. The successor organisation, the International Alliance for Responsible Drinking has operated differently and has managed to almost entirely avoid attracting critical scientific attention. Achieving a fuller understanding of ICAP may help orientate further study of the alcohol industry's ongoing involvement in science, and in policymaking. We are unlikely to have another resource of the kind we have in the tobacco documents library put into the public domain any time soon; therefore, it is vital to continue to pay careful attention to industry activities on (and beyond) the fringes of peer‐reviewed science.

## AUTHOR CONTRIBUTIONS

Andrew Bartlett was the lead author of this article. Jim McCambridge contributed throughout the drafting process. Jack Garry led the initial document search and contributed to the drafting process. All authors read and reviewed the final article.

## FUNDING INFORMATION

This study was funded by the Wellcome Trust, via an Investigator Award to Jim McCambridge (200321/Z/15/Z).

## CONFLICT OF INTEREST STATEMENT

The authors declare that they have no competing interests.

## ETHICS STATEMENT

Not applicable.

## Data Availability

This article is an analysis of the tobacco documents archive and publicly available books.
